# Healthy Adult LDL-C Bears Reverse Association with Serum IL-17A Levels

**DOI:** 10.2174/2213988501812010001

**Published:** 2018-06-29

**Authors:** Azam Roohi, Mina Tabrizi, Mehdi Yaseri, Fereshteh Mir Mohammadrezaei, Behrouz Nikbin

**Affiliations:** 1 Department of Immunology, School of Public Health,Tehran University of Medical Sciences,Tehran,Iran.; 2 Department of Medical Genetics, School of Medicine,Tehran University of Medical Sciences,Tehran,Iran; 3 Department of Epidemiology and Biostatistics, School of Public Health,Tehran University of Medical Sciences,Tehran,Iran; 4 Department of Biology, Faculty of Science, University of Mazandaran, Mazandaran, Babolsar,Iran; 5 Department of Immunology, School of Medicine,Tehran University of Medical Sciences,Tehran,Iran

**Keywords:** Low-density lipoprotein cholesterol, LDL-C, IL-17A, Atherosclerosis, Inflammation

## Abstract

**Background:**

Hypercholesterolemia is a modifiable risk factor in atherosclerosis with a complex association with inflammation.

**Objective:**

In the present study, the association between low-density lipoprotein cholesterol (LDL-C) and interleukin 17A (IL-17A), as an inflammatory cytokine, was investigated. In addition to IL-17A, serum levels of interleukin 23 (IL-23) and transforming growth factor β (TGF-β), as effective cytokines in T helper 17 cell (Th17) development, were also determined.

**Method:**

Cytokine levels were measured using enzyme-linked immunosorbent assay (ELISA) in healthy subjects with LDL-C<130 versus LDL-C=>130 mg/dL.

**Results:**

Although IL-17A is an inflammatory cytokine and a positive association between its levels and LDL-C is expected, the data obtained in this study provide support for a reverse association (*p*<0.05).

**Conclusion:**

Inflammation plays a major role in atherosclerosis development; however, various inflammatory components involved in atherosclerosis assert their own unique association with hypercholesterolemia.

## 1 INTRODUCTION

Atherosclerosis is one of the most common causes of cardiovascular disease. It begins early in life, making primary prevention efforts necessary from childhood [1]. Thus, there is increasing emphasis on preventing atherosclerosis by modifying risk factors. Dyslipidemia is one of the nine potentially modifiable factors accounting for over 90% of the population-attributable risk for a first Myocardial Infarction (MI) as demonstrated by the INTERHEART study, the first study to systematically examine the determinants of vascular disease in 52 countries [2]. Extensive research indicates elevated LDL-C cholesterol is a major cause of Coronary Heart Disease (CHD). Clinical trials have shown that LDL-C-lowering therapy reduces the risk for CHD. Therefore, initiation of treatment is based on LDL-C levels and target of treatment is LDL-C [3, 4]. Nonetheless, in a cohort, it was observed that almost 70% of hospitalized patients, due to coronary artery disease, had LDL-C levels lower than 100 mg/dL upon admission [5].

Undoubtedly there is a complex association between lipid metabolism, atherosclerosis and inflammation [6]. Insights into atherosclerosis as an inflammatory disease offer the opportunity to find new risk predictors and develop novel therapeutic strategies targeting the inflammatory component of the disease or more preferably directly targeting the culprit initiating and perpetuating the inflammatory process. Chronic tissue inflammation translates into inflammatory cytokines in the system. Cytokine secretion and activation can result in damage to the vascular endothelium priming the environment towards atherogenesis or causing existing plaque rupture, thrombosis, and even acute ischemic symptoms [7]. Lipids can also modulate the inflammatory response, but inflammatory signaling can significantly alter lipid metabolism in the context of atherosclerosis [8]. Anti-inflammatory and immunosuppressive mechanisms inhibit atherosclerosis and may be attractive targets for disease prevention and/or treatment [9]. Among inflammatory mediators, raised plasma levels of C-Reactive Protein (CRP) is associated with increased risk of coronary heart diseases [10]. In addition, IL-6 and IL-1β are CRP upstream mediators associated with cardiovascular events [11]. IL-17A is signature cytokine of Th17 cells associated with inflammatory and autoimmune diseases [12], and its association with atherosclerosis is still controversial. Th17 differentiation is promoted by a set of cytokines which include TGF-β. TGF-β induces the RAR-related orphan receptor C (RORc) as the master transcription factor to direct the Th17 differentiation program in humans [13]. To the best of our knowledge, this is the first study to measure IL-17A, IL-23 and TGF-β along with LDL-C in healthy adults. The aim of this study was to investigate any possible association between serum levels of TGF-β, IL-23, IL-17A and LDL-C in healthy people with increased levels of LDL-C.

## 2 MATERIALS AND METHODS

### 2.1 Participants

Study samples were collected from Jul. to Sep. 2011 from the “Diabetes Screening Project” conducted on a volunteer population at Tehran’s Farmanfarmaian Health Center. In addition, a few subjects were recruited from healthy workers referred to the same center for their periodical medical tests in compliance with the Occupational Medicine Rules of Iran. After gaining approval from the ethics committee of TUMS and obtaining informed consents, background information such as weight, height and age were recorded. Subjects with known disease were excluded. Serum samples of the remaining subjects were collected and stored at -20 °C and biochemical tests were conducted for 120 people.

Using autoanalyzer (Hitachi 902, Japan) Fasting Blood Glucose (FBS), total cholesterol, High- Density Lipoprotein Cholesterol (HDL-C) and triglyceride (TG) were measured. LDL-C was measured using commercial kits (Parsazmun, Iran).

After conducting biochemical tests, all subjects with FBS =>105 mg/dL were also excluded. Finally, 74 subjects (42 males and 32 females) were selected for the present study. According to the serum LDL-C levels, these subjects were divided into two groups (*i.e.* people with LDL-C =>130 mg/dL or LDL-C <130 mg/dL) [3].

### 2.2 Cytokine Assays

Serum concentrations of IL-17A, IL-23 and TGF-β were measured using commercially available enzyme-linked immunosorbent assay kits (eBioscience ELISA kits,USA) as described previously [14]. Sensitivity of the kits was follows: IL-17A; 4 pg/ml, IL-23; 15 pg/ml and TGF-β; 60 pg/ml. In brief, Maxisorp immuno plates (Nunc, Denmark) were coated with monoclonal antibodies (mAb) specific for IL-17A, IL-23 or TGF-β. Then, serum samples and standards were added and serum cytokines were detected using biotinylated mAb specific for IL-17A, IL-23 or TGF-β followed by addition of streptavidin-horseradish peroxidase and color development. Absorbance was read at 450 nm. Using standard curves, the values were expressed in picogram per milliliter.

### 2.3 Statistical Analysis

All data are presented as means ± SD. To compare groups for continuous variables, Mann-Whitney test was used. To determine the association of IL-17A, IL-23 and TGF-β with other parameters, Pearson and Spearman correlation tests were conducted. In all analyses, *p* values <0.05 were considered significant. All statistics were done using SPSS for windows version 19.

## 3 RESULTS

This study was conducted to investigate any association between IL-17A, IL-23, TGF-β and LDL-C in people with LDL-C higher than normal range (130 mg/dL). Table **1** shows the demographic and clinical characteristics of the study subjects.

**Fig. (1):**
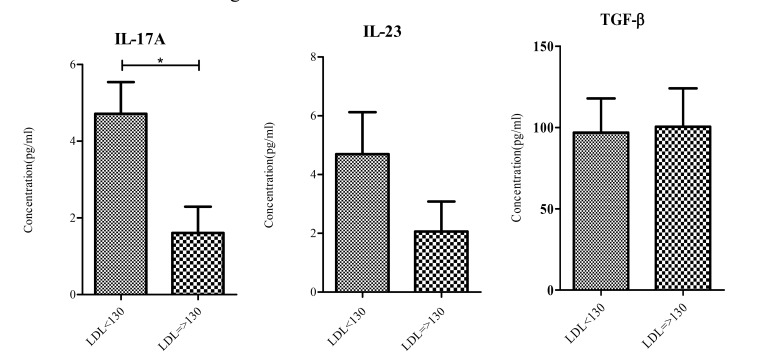
Serum IL-17A, IL-23 and TGF-β levels in study subjects. IL-17A levels were significantly lower in subject with LDL-C => 130 *p value < 0.05.

Statistical analysis showed a significant difference between serum IL-17A levels in subjects with LDL =>130 and those who had LDL-C<130. No significant difference was found between two groups in terms of IL-23 and TGF-β serum levels. Results are summarized in Fig. **1** and Table **2** .

Using Pearson correlation coefficients, relations between LDL-C and the three mentioned cytokines were analyzed. Results of the analysis indicated no relation between these cytokines and LDL-C while data provide support for a correlation between IL-17A levels and IL-23 levels in serum as was expected (r = 0.771).

## 4 DISCUSSION

During acute infections, hypertriglyceridemia can occur due to tumor necrosis factor α (TNF-α) effects on hepatic lipogenesis and lipoprotein lipase. In addition, noticeable concentrations of cholesterol are needed for proliferation of activated T cells which requires cholesterol mobilization. This relationship between metabolism and the immune system is multi-faceted in chronic diseases such as rheumatoid arthritis and atherosclerosis [15] in which a vicious cycle links hypercholesterolemia and inflammation. LDL-C is the leading atherogenic cholesterol carrier lipoprotein. Elevation of plasma levels of LDL-C promotes its retention in the arterial intima. Various modified forms of LDL-C, such as oxidized or aggregated LDL-C can also bind scavenger and Toll-like receptors on macrophages or be taken up *via* micropinocytosis or phagocytosis by macrophages [16]. In addition to macrophages, lipids can activate endothelial cells with the capability to express adhesion molecules, and, of course, adhesion molecule expression is enhanced by turbulent blood flow [17]. Upregulation of adhesion molecules and chemokine production culminate in the recruitment of other immune cells including T and B cells. Indeed, in advanced atherosclerosis, ectopic lymphoid organs are generated [18]. Considering all these events, it seems reasonable to expect an association between LDL-C levels and inflammatory mediators.

In the present study, the existence of a positive association between LDL-C and serum IL-17A level, as an inflammatory marker, was hypothesized. We measured serum levels of IL-17A, IL-23 and TGF-β in healthy adults who demonstrated LDL-C levels higher than 130 mg/dL. Our aim was to find an association between LDL-C and IL-17A as an inflammatory cytokine in atheroma development before any disease observation. The serum levels of IL-23 and TGF-β, as effective cytokines in the development of Th17 cells, were also determined. The results indicated that the levels of IL-17A demonstrated reverse association with LDL-C.

Physiologically, development of atherosclerotic plaques is a lengthy and complicated process in which the immune system assumes a Janus-faced identity [19]. As mentioned above, it seems that an interplay between modified LDL-C or associated LDL-C molecules and the innate immune system triggers plaque formation followed by acquired immune system activation. Macrophages, as key cellular players in plaque formation, gain various phenotypes and characteristics affected by lipids, cytokines, irons and senesced cells [20]. These cells modulate atheroma progression in part *via* secretion of anti-atherosclerotic (TGF-β, IL-10) and/or pro-atherosclerotic mediators (IL-6, TNF-α) [21].

In addition to the innate immune system, there are many reports regarding opposing roles played by different T cell subsets in atherosclerosis. Th1 cells produce interferon γ (IFN-γ). This cytokine induces foam cell formation, macrophage activation and prohibits smooth muscle cell proliferation and differentiation which results in plaque destabilization [22]. As it is expected, natural regulatory T cells (T_Reg_) show atheroprotective effects via TGF-β and IL-10 production [23]. Role of Th17 and its signature cytokine, IL-17A, in atherosclerosis is controversial [24, 25]. In a review of recent literature, Yu *et al*. concluded that IL-17A seems to have atherogenic properties [26]. In a research conducted on apolipoprotein E-deficient mice, deficiency of IL-17A resulted in accelerated formation of unstable atherosclerotic plaques [27]. Gistera *et al*. observed that TGF-β can stabilize plaques *via* an IL-17A-dependent pathway [28] while it is reported that blockade of IL-17A resulted in decreased lesion size and stabilization [29]. Thus, IL-17A may be serving a dual function depending on the mechanism that is controlling it. If IL-17A is activated by TGF-β, it stabilizes plaques. On the other hand, IL-17A suppression by a yet unknown mechanism, leads to lesion size reduction and stabilization.

In addition to the immune system, there are elements which exert counteracting effects on inflammation promotion during atheroma formation. Interestingly, cholesterol itself plays contradictory roles. Accumulation of cholesterol inside macrophages leads to increased activation of Toll-like receptors and inflammasome formation and also production of more monocytes and neutrophils. On the other hand, cholesterol can activate a heterodimeric transcription factor, liver X receptor (LXR)–retinoid X receptor (RXR), with anti-inflammatory effects [30]. It is not counterintuitive to observe protective mechanisms being activated in response to assaults on normal human physiology (pathology).

Surendar **et al.** have unexpectedly found a decrease of IL-17A levels in subjects with metabolic syndrome (MS) abnormalities [31]. Metabolic syndrome is a group of CHD and diabetes risk factors including abnormal levels of cholesterol and TG. In MS inflammatory pathways are also activated [32].In Surendar study, subjects with MS had TG levels which were significantly higher than the control group while there was no significant difference between LDL-C levels in their case and control groups [31]. It seems that our findings regarding IL17-A are in line with the study conducted by Surendar *et al*. In the present study, the TG level along with LDL-C levels showed significant differences between the two study groups. It was significantly higher in subjects with LDL-C=>130(*p* <0.001). It would be intriguing to study any possible relationship between TG and IL-17A. Since IL-23 is a major Th17 cell inducer and there is a positive correlation between IL-23 and IL-17A levels, it seems reasonable to observe a similar positive correlation between IL-23 and IL-17A levels in plasma. In the present study, we observed rise in IL-23 levels corresponded to a rise in IL-17A levels in study subjects sera.

In the present study, TGF-β levels were measured because TGF-β is a cytokine required for Th17A development [13], but no significant difference between the two study groups was observed. TGF-β affects different cell types involved in atheroma development including macrophages, endothelial cells and vascular smooth muscle cells. The major effect of TGF-β on macrophages is anti-atherogenic. However, function of this cytokine in atherosclerosis progression is not clear due to its roles in fibrosis promotion and inhibition of endothelial regeneration [33]. Recently conducted research has revealed a mutual interplay between TGF-β and lipids. The TGF-β signaling pathway has been shown to be dysregulated by cholesterol in a variety of cell lines [34]. In atherosclerotic mice, researchers have found positive correlation between cholesterol and TGF-β levels in plasma. It was speculated that elevated levels of TGF-β may neutralize pro-inflammatory effects of hypercholesterolemia [35]. In patients with advanced atherosclerosis, reduced plasma levels of this cytokine have been reported [36] meaning that different concentrations of this cytokine during atherosclerosis progression may be associated with disease stage [37].

## CONCLUSION

This is a cross-sectional study on healthy adults with elevated levels of LDL-C who may or may not develop atherosclerotic plaques in the future. No causal relation may be found between measured cytokines and atherosclerosis incidence but any predictable associations can allow planning of interventions to prevent atherosclerotic diseases. Recently, the application of canakinumab, a monoclonal antibody against IL-1β, effectively reduced the rate of cardiovascular events [38]. Meanwhile, further studies of similar nature may allow scientists to shed more light on the lengthy and complicated mechanism of atheroma formation and discovery of related inflammatory/anti-inflammatory predictors.

## Figures and Tables

**Table 1:** Demographic and clinical characteristics of participants.

	LDL-C < 130 mg/dL	LDL-C = > 130 mg/dL	*p-*value
	(n = 54)	(n = 20)	
Age (year)	35.9 ± 10.8	44.5 ± 12.6	0.010‡
Sex (F/M)	27/27	5/15	0.054
BMI(kg/m^2^)	25.3 ± 4.4	25.8 ± 3.2	0.484‡
FBS(mg/dL)	86.8 ± 7.4	85.8 ± 7.8	0.555‡
Chol(mg/dL)	181.2 ± 30.8	239.4 ± 33	0.000‡
TG(mg/dL)	122.8 ± 69.7	185.6 ± 70.9	0.000‡
HDL-C(mg/dL)	52.5 ± 10	50.3 ± 10.6	0.303‡
LDL-C(mg/dL)	97.6 ± 18.7	154.8 ± 21	0.000‡

‡ Based on Mann-Whitney test

M:male, F: female BMI: body mass index, FBS: fasting blood glucose, Chol: cholesterol

**Table 2:** – Cytokine serum levels in subjects with LDL-C => 130 and LDL-C<130.

LDL-C<130 LDL-C >= 130					
	Mean ± SD	Median (Range)	Mean ± SD	Median (Range)	*p*-value‡
IL-17A	4.72 ± 5.99	1.97 (0 to 28.48)	1.43 ± 3	0.31 (0 to 12.94)	0.016
IL-23	8.946 ± 33.462	0 (0 to 238.953)	2.167 ± 4.757	0 (0 to 17.67)	0.526
TGF-β	105.347 ± 156.143	66.175 (0 to 934.014)	82.271 ± 101.133	54.355 (0 to 409.427)	0.669

‡ Based on Mann-Whitney test.

## LIST OF ABBREVIATIONS

LDL-C: = Low- density lipoprotein cholesterol
IL-17A: = Interleukin 17A
IL-23: = Interleukin 23
TGF- β: = Transforming growth factor β
Th17: = T helper 17
ELISA: = Enzyme-linked immunosorbent assay
MI: = Myocardial infarction
CHD: = Coronary heart disease
CRP: = C-reactive protein
RORc: = RAR-related orphan receptor C
FBS: = Fasting blood glucose
HDL-C: = High- density lipoprotein cholesterol
TG: = Triglyceride
mAb: = Monoclonal antibodies
TNF-α: = Tumor necrosis factor α
IFN γ: = Interferon γ
Treg: = Regulatory T cells
LXR: = Liver X receptor
RXR: = Retinoid X receptor
MS: = Metabolic syndrome
